# User-Centered Formative Evaluation of Cognitive Rehabilitation Software: Cognitive Walkthrough and System Usability Scale Study

**DOI:** 10.2196/75805

**Published:** 2025-11-18

**Authors:** Seojin Hong, Hyun Choi, Hyosun Kweon

**Affiliations:** 1 National Rehabilitation Center Seoul, Gangbuk-gu Republic of Korea

**Keywords:** usability evaluation, formative evaluation, IEC 62366-1, cognitive walkthrough, System Usability Scale, cognitive rehabilitation software, human factors

## Abstract

**Background:**

The usability of software as a medical device plays a critical role in ensuring patient safety, clinical effectiveness, and treatment adherence. Particularly in tablet-based cognitive rehabilitation software, poor usability can increase the risk of user errors, cognitive overload, and low engagement, which may diminish therapeutic outcomes. Despite the growing integration of cognitive rehabilitation software in clinical settings, few studies have systematically evaluated its usability using structured human factor methodologies.

**Objective:**

This study aimed to conduct a formative evaluation of a tablet-based cognitive rehabilitation software by identifying potential user errors, interface design issues, and opportunities for improving the user experience and user interface (UI).

**Methods:**

Following the International Electrotechnical Commission (IEC) 62366-1 framework for usability engineering, a formative evaluation was conducted using a combination of cognitive walkthrough (CW) and the System Usability Scale (SUS). CW was used to identify interaction breakdowns and potential use-related problems through scenario-based expert evaluation. The SUS was administered to quantitatively assess perceived usability. Five occupational therapists with experience in cognitive rehabilitation participated in the formative evaluation.

**Results:**

A total of 14 usability issues were identified across 6 task scenarios. Common problems included difficulty locating buttons, insufficient feedback during task completion, and inconsistent UI behavior. Key improvement areas included keypad layout, visibility of task progress, and accessibility of help content. The mean SUS score was 73.5 (SD 11.4), indicating an acceptable usability level (grade of B–). The lowest scores were related to system complexity and interface integration, suggesting a need to optimize UI flow and content hierarchy.

**Conclusions:**

The combined use of CW and the SUS enabled the identification of both objective and perceived usability challenges in the cognitive rehabilitation software. Findings emphasize the importance of user-centered design and formative evaluation in the early stages of software development. Recommendations such as improving information visibility, reducing unnecessary steps, and enhancing system feedback may contribute to safer and more effective digital cognitive interventions. However, given the small and homogeneous participant sample and the controlled test environment, these findings should be interpreted as preliminary. This study highlights the practical application of the IEC 62366-1 framework in evaluating software as a medical device and demonstrates its utility in guiding iterative user experience and UI improvements in cognitive rehabilitation contexts.

## Introduction

With advancements in digital technology, the adoption of computer-based cognitive training is expanding in the fields of medicine and rehabilitation [1,2]. Specifically, tablet- and computer-based cognitive rehabilitation software is being used as an effective rehabilitation tool due to its high accessibility and intuitive touch screen interface [3,4]. The primary aim of cognitive rehabilitation software is to enhance patients’ cognitive functions through iterative training. However, simplistic program designs alone do not guarantee clinical efficacy. For the effective utility of such software, evaluating the user interface (UI) and user experience (UX) design and usability—factors that shape the experiences of its intended users, such as occupational therapists—is crucial. This usability evaluation is strongly linked to treatment adherence and clinical efficacy [5].

Usability evaluation of medical devices and digital rehabilitation software is critical for reducing therapists’ workload, increasing treatment engagement, and ensuring the clinical reliability of the software [6]. In the context of cognitive rehabilitation software, poor usability may impose unnecessary cognitive demands on occupational therapists—such as navigating convoluted menus or locating key functions—which can slow down therapy sessions and distract from patient engagement. For instance, Levac and Miller [7] reported that even experienced therapists encountered usability barriers during a tablet-based rehabilitation tool study, identifying numerous navigational failures that disrupted session flow and required repeated assistance during task completion. Moreover, in analogous rehabilitation contexts—such as robotic exoskeleton use—studies have demonstrated that higher usability and lower cognitive workload are associated with greater therapeutic benefit and user efficiency [8]. These findings suggest that usability deficits in cognitive rehabilitation tools can directly degrade work efficiency and potentially undermine clinical and patient outcomes in real-world settings [9].

In particular, for software functioning as a medical device, it is critical to ensure that users can interact with the system correctly and efficiently in real-world clinical contexts. To address this, the International Electrotechnical Commission (IEC) 62366-1 standard outlines a structured usability engineering process for medical devices, emphasizing the prevention of use-related errors and the enhancement of user-system interaction safety [10]. This standard advocates for the incorporation of formative evaluation during early design stages to identify and rectify potential design flaws before final implementation. Formative evaluations, as defined by the standard, aim to detect use errors and interface issues that could compromise task performance, especially in cognitively demanding contexts. In pediatric cognitive rehabilitation, where users’ cognitive abilities and task comprehension may vary widely, adopting such structured usability engineering is essential to ensure both therapeutic effectiveness and user safety. Despite this, few empirical studies have applied the IEC 62366-1 framework specifically to the evaluation of cognitive rehabilitation software, indicating a significant gap in the empirical evidence supporting standard-compliant usability practices in this domain.

Among formative evaluation methods, the cognitive walkthrough (CW) is an effective evaluation method for analyzing design errors that may occur during system navigation, and it also serves as a common methodology for evaluating the intuitiveness of UX and UI design during the inception of medical device development [11]. The System Usability Scale (SUS) is a quantitative tool used to evaluate the overall usability of medical software and is also useful for objectively measuring UX [12].

Previous studies have primarily focused on evaluating the clinical efficacy and treatment outcomes of computerized cognitive rehabilitation software [13,14], with few studies assessing usability to improve UI and UX design. The intuitiveness and ease of the UI and UX in medical device software directly affect clinical applicability and treatment adherence, whereas poorly designed interfaces increase user errors, reducing treatment effectiveness.

Therefore, this study aimed to evaluate the usability of a tablet-based cognitive rehabilitation software by identifying use-related problems and UI and UX design issues. Using CW and the SUS, we assessed the experiences of occupational therapists, the primary users of this software, and proposed design improvement strategies aimed at enhancing the intuitiveness, accessibility, and overall clinical applicability of cognitive rehabilitation software.

## Methods

### Study Design

This study was conducted as a formative evaluation of a prototype tablet-based cognitive rehabilitation software for children with cognitive impairment. The CW and the SUS were used with occupational therapists to identify use-related problems and gather UX insights to inform subsequent interface improvements.

### Cognitive Rehabilitation Software

The cognitive rehabilitation software used in this study was developed for use in occupational therapy to enhance cognitive function, learning, and development among patients with cognitive impairments. This product comprises 12 subprograms, including 7 structured programs arranged by difficulty level (puzzles, finding hidden objects, finding animals, finding the same pictures, finding rules, memorizing, and guessing the sound) and 5 nonstructured programs (daily life play, drawing along, balloon play, farm play, and guessing objects). In addition, a cognitive screening evaluation, result checking, use guidelines, and instructions are available. Specifically, the software assesses patients’ cognitive status through an initial screening evaluation and subsequently recommends individualized cognitive therapy programs based on the obtained results. Occupational therapists design and monitor personalized rehabilitation treatment sessions, and analytical outcomes can be reviewed upon completion of each session.

The main UI consisted of a home dashboard displaying program categories, patient management tools, and progress-tracking features. Each rehabilitation module featured consistent navigation patterns, including start and end buttons, difficulty selection, and progress indicators. Due to intellectual property and confidentiality restrictions requested by the manufacturer, the detailed UI design and proprietary elements of the software could not be disclosed. However, the evaluation was conducted using the actual commercial prototype provided by the manufacturer.

The recommended software specifications include an Android 13–based operating system equipped with either a Samsung Exynos 1380 or Qualcomm Snapdragon 865 processor. In this study, the Samsung Galaxy Tab S6 Lite was used.

### Participants

The formative evaluation was conducted in accordance with the IEC 62366-1 standard, which emphasizes formative evaluation during the early stages of medical device development. This approach typically involves a limited number of representative users or domain experts to iteratively identify potential use-related problems and interface design issues. According to the IEC 62366-1 and previous usability research [11,15], a sample of 3 to 8 participants is generally sufficient to detect most usability problems during formative testing. Accordingly, 5 occupational therapists affiliated with the Korea National Rehabilitation Center (NRC)—the primary users of cognitive rehabilitation software—participated in this evaluation. While appropriate for formative evaluation, this narrow sample from a single institution excludes other relevant user groups such as patients and caregivers, thereby limiting the representativeness of the findings. Nonetheless, their participation enabled in-depth identification of task flow difficulties, interface-related usability issues, and UX improvement needs. Expert-based formative evaluation at this stage is particularly important in the context of medical software as it supports early detection of design flaws that could lead to clinical use errors, thereby contributing to risk mitigation and overall safety.

The formative evaluation participants (N=5) were all licensed occupational therapists with previous experience using medical devices for cognitive rehabilitation. Inclusion criteria required sufficient proficiency in both Korean and English to ensure comprehension of the software’s terminology and interface instructions. Individuals without previous experience in cognitive rehabilitation therapy were excluded. All participants voluntarily provided written informed consent after receiving a detailed explanation of the study’s objectives and procedures and their rights as participants.

The evaluators included 3 usability researchers from the NRC: 1 facilitator and 2 observers. The facilitator was responsible for the overall execution of the evaluation, including orientation, task instruction delivery, informed consent collection, and administration of pre-evaluation surveys and postevaluation interviews. The observers were tasked with monitoring and documenting each participant’s task performance and behavioral responses throughout the formative evaluations.

### Formative Evaluation Protocol

This study conducted a formative evaluation following the UI validation procedures outlined in the IEC 62366-1 standard, which emphasizes the early identification and mitigation of use-related risks in medical device software. The evaluation was carried out at the NRC using a combination of CW and the SUS.

CW is a scenario-based expert method that examines user interaction flows to detect potential usability issues within the UI. It is particularly well suited for evaluating systems with high cognitive demands, such as pediatric cognitive rehabilitation software. The SUS, a standardized 10-item questionnaire, was used to quantitatively assess users’ subjective perceptions of usability. The integration of CW and the SUS enabled a comprehensive evaluation of both objective interaction challenges and subjective UX during early-stage development.

**Figure 1:**
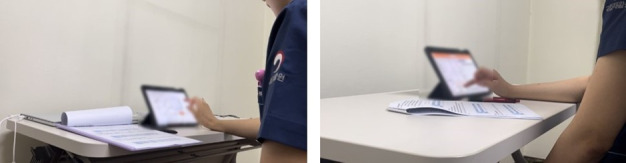
Performance of task scenarios by evaluation participants.

Each evaluation session lasted approximately 50 minutes per participant (Table 1). The facilitator provided participants with an overview of the study objectives and procedures, anonymized product information, and usability testing process. General demographic characteristics and previous experience with cognitive rehabilitation devices were collected, after which participants performed tasks based on predefined software scenarios. Throughout the evaluation, an observer recorded task performance and behavioral responses. Upon completion of the tasks, participants completed the SUS questionnaire to assess the overall usability of the software (Figure 1).

**Table 1:** Formative evaluation procedures conducted for 50 minutes under the guidance of a facilitator.

Stage	Details	Time (min)
Orientation	The facilitator introduces the concept of formative evaluation to participants who are unfamiliar with it. The facilitator explains the target product, purpose, overview, methodology, and other relevant aspects of the formative evaluation to the participant.	5
Guidance on consent	The facilitator provides the participant with detailed instructions regarding recording and transcription procedures. The facilitator ensures that the participant fully comprehends the evaluation content and voluntarily provides written consent to take part in the formative evaluation.	5
Cognitive walkthrough	The facilitator conducts a cognitive walkthrough following a predefined script and product use instructions. The participant operates the medical device under the facilitator’s guidance, verbalizes their thoughts during task performance, and provides feedback on their experiences. The observer documents and analyzes the participant’s responses during task performance.	30
Administration of the SUS^a^	After completing the cognitive walkthrough, the facilitator administers the SUS to the participant to assess the usability of the user interface.	10

^a^SUS: System Usability Scale.

Before conducting the formative evaluation, researchers reviewed the software’s intended use, functional specifications, and workflow documentation included in the usability engineering file provided by the manufacturer. This documentation described the core business processes of the software—such as patient registration, cognitive assessment, training program delivery, and result analysis. The task scenarios were explicitly mapped to these clinical workflows to ensure that the evaluation reflected real-world therapist operations and covered the complete use process. All task scenarios were developed based on the scenario construction procedures outlined in the IEC 62366-1 standard and the usability engineering file. Although no formal pilot test was conducted with intended users, the sequence of actions and task objectives was validated through internal simulation by usability researchers to ensure logical consistency, alignment with the intended use, and appropriateness of user-interface interaction flows. This approach, consistent with formative evaluation standards, was considered sufficient for verifying scenario validity during the early design stage.

The formative evaluation environment simulated a real-world occupational therapy room. To maintain environmental consistency, the test room was premeasured and adjusted to specific parameters: illumination at 316 lx, ambient noise at 28 dBA, room temperature at 22.8 °C, and relative humidity at 67%.

### CW Methodology

CW is a commonly used quantitative evaluation method to assess the cognitive load of users during system navigation and task performance [11,16]. To evaluate whether the participants could effectively navigate the software, the facilitator provided the task scenarios without previous training. The evaluation participants navigated the software according to the task scenarios provided and performed the major tasks. The observer and recorder documented UI and UX issues and user errors encountered during the evaluation process.

CW was used to analyze major usability issues that might arise as users navigated the software. For this, the evaluation involved a total of 8 major tasks divided into 17 subtasks, including starting the software, performing a cognitive screening evaluation, setting patient accounts, and selecting and running rehabilitation content. The eight evaluation tasks were (1) checking the power, (2) registering patient information, (3) patient evaluation, (4) checking the user manual, (5) running the program 1, (6) running the program 2, (7) analyzing the results, and (8) exiting the program (Table 2).

**Table 2:** Task scenarios.

Task	Subtask
Checking the power (task 1)	The therapist turns the power on in the tablet screen and runs the cognitive rehabilitation software (subtask 1.1).
Patient information registration (task 2)	The therapist generates patient information (subtask 2.1). The therapist selects name for the patient (subtask 2.2).
Patient evaluation (task 3)	The therapist performs a cognitive development screening evaluation on the patient (subtask 3.1). The therapist ends the cognitive development screening evaluation (subtask 3.2).
Checking the user manual (task 4)	The therapist checks the guidelines and explanations for the software programs on finding hidden objects and memory (subtask 4.1).
Running the program 1 (task 5)	The therapist sets the settings for the program on finding hidden objects and applies the program on the patient (subtask 5.1). The therapist adjusts the difficulty level of the program on finding hidden objects and applies the program on the patient (subtask 5.2). The therapist provides hints to the patient (select the button within the program; subtask 5.3). The therapist ends the program on finding hidden objects and returns to the main screen (subtask 5.4).
Running the program 2 (task 6)	The therapist sets the settings for the memory program and applies the program on the patient (subtask 6.1). The therapist ends the memory program and returns to the main screen (subtask 6.2).
Analysis of results (task 7)	The therapist checks the user information (subtask 7.1). Check the results of “Evaluation participation date” (subtask 7.2). The therapist checks the specific results for each date (programs applied, levels performed, number of items, correct answer rate, response time, and total performance time; subtask 7.3). The therapist checks the comprehensive analysis (performance, memory, concentration, hand-eye coordination, language skill, and visual perception; subtask 7.4).
Exit (task 8)	Exit the cognitive rehabilitation software (subtask 8.1).

The observer monitored the evaluation participants as they performed the subtasks, documenting and analyzing the following: (1) whether the user knew what they needed to do for each task (response options: yes or no), (2) whether the user performed and completed the task properly (response options: yes or no), and (3) task completion status (response options: yes or no) [17,18]. In addition, the reactions and verbal opinions of the participants were recorded.

### SUS Questionnaire

The SUS is a quantitative evaluation tool used to assess how users perceive system usability [12]. The SUS comprises 10 items rated on a Likert scale (1=“strongly disagree”; 5=“strongly agree”; Table 3). The total score is converted to a range from 0 to 100 points to evaluate the overall system usability. Moreover, the SUS scores are assigned a letter grade from F (0-60 points) to A (91-100 points) according to a standardized grading system [19,20].

**Table 3:** System Usability Scale questionnaire item breakdown.

Item category	Statement
Utility	“I think that I would like to use this system frequently.”
Complexity	“I found the system unnecessarily complex.”
Simplicity	“I thought the system was easy to use.”
Professionalism (technician support)	“I think that I would need the support of a technical person to be able to use this system.”
Integration	“I found the various functions in the system were well integrated.”
Unity	“I thought there was too much inconsistency in this system.”
Learnability	“I would imagine that most people would learn to use this system very quickly.”
Convenience	“I found the system very cumbersome to use.”
Satisfaction	“I felt very confident using the system.”
Professionalism (previous learning)	“I needed to learn a lot of things before I could get going with this system.”

### Data Collection and Analysis

CW was used to analyze major usability issues that might occur during software navigation. User reactions and opinions, comprising qualitative data collected through CW, were transcribed, managed, and coded using Microsoft Excel based on the observer and recorder records. SUS evaluation was used to quantitatively assess the overall software UX. The mean and SD of the SUS scores were calculated and interpreted as letter grades (A to F) according to the grading system [19].

### Ethical Considerations

This study was approved by the institutional review board of the NRC (NRC-2024-04-020). All participants provided voluntary consent to take part after receiving a comprehensive explanation of the study objectives and methods. No compensation was provided for participating in the study. For the protection of personal information and the confidentiality of the participants, all data were anonymized.

## Results

### Evaluation of Participant Information

The evaluation participants comprised 5 occupational therapists with 3 years and 2 months to 15 years and 4 months of experience. Furthermore, the therapists had 2 to 12 years of experience using similar medical devices or computerized cognitive programs, such as ComCog (Neofect), RAPAEL (Neofect), and RehaCom (HASOMED GmbH; Table 4).

**Table 4:** General characteristics of the participants.

ID	Sex	Experience (y)	Experience with similar medical devices			
			Used them before	Model name	Experience of use (y)	Frequency of use

P1	Female	≥6	Yes	RAPAEL	2	2-3 times per mo
P2	Female	≥3	Yes	ComCog and RAPAEL	3	1-2 times per wk
P3	Male	12	Yes	ComCog and RehaCom	12	2-3 times per d
P4	Female	≥4	Yes	ComCog and RAPAEL	4	3 times per wk
P5	Male	≥15	Yes	ComCog, RAPAEL, and RehaCom	10	1 time per wk

### CW Results

During the CW, which involved observing task performance by the participants based on the task scenarios, frequent typing errors were noted during patient registration (task 2) and patient information generation (subtask 2.1) due to the small size of the on-screen keypad. Participants also experienced inconveniences when checking certain information, as the keypad obscured a section of the patient information input. Furthermore, they experienced difficulty finding the delete button and delays when looking for the button to change to English to input information in English.

To perform the cognitive development screening evaluation (subtask 3.1) within the patient evaluation task (task 3), some participants appeared confused because the evaluation began without prior instruction or explanation. In particular, although the puzzle task required a tapping gesture, several participants attempted to use a dragging gesture, likely because of their previous experience with similar interfaces. This reflected a mismatch between the user’s mental model and the intended interaction design. To complete the cognitive development screening evaluation (subtask 3.2), participants were required to press the “Next Item” button as a separate “End” button was not provided. However, evaluation participants mistakenly pressed the “Back” button to end the evaluation, which led to the loss of evaluation results. This issue made it impossible to check the cognitive development results, impacting the running of the program.

To check the user manual (task 4), most users appeared not to recognize the “What is cognitive rehabilitation software?” icon as the button for the software user manual.

To adjust the difficulty (subtask 5.2) within the task of running the program (task 5), the selection of the program stage appeared on the program screen, whereas the selection of the number of items appeared via a modal pop-up. Users made errors by pressing the “Run” button without recognizing the stage selection screen when selecting the stage and number of items. Regarding giving hints (subtask 5.3), 60% (3/5) of the evaluation participants did not recognize the question mark icon as the hint button and instead provided verbal hints, resulting in errors.

To check the results of the “Evaluation participation date” (subtask 7.2) within the result analysis task (task 7), 40% (2/5) of the participants did not end the cognitive development screening evaluation as indicated in the user manual. Consequently, the evaluation results were not saved, and thus, the list of programs performed and performance results could not be confirmed. Moreover, 40% (2/5) of the participants selected the detailed program results (individual training session outcomes) instead of the screening evaluation performance results (summary results from the initial cognitive development screening). Regarding checking the specific results for each date (subtask 7.3), 40% (2/5) of the participants did not complete the cognitive development screening evaluation. Consequently, specific results for each date could not be found and confirmed. Moreover, 40% (2/5) of the participants could not find the icon for specific results by date, and therefore, they were unable to perform this subtask. To check the performance level of the patient for the cognitive domain (subtask 7.4), the correct icon was selected, but because the cognitive development screening evaluation results were not saved, the cognitive ability graph was not provided and could not be confirmed.

### SUS Results

**Figure 2:**
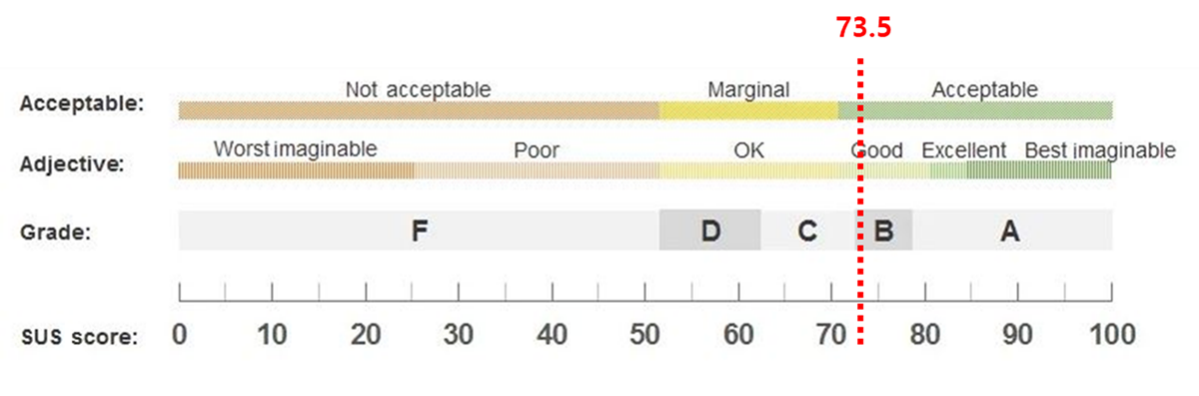
System Usability Scale (SUS) results.

The mean SUS score was 73.5 (SD 7.2) points, equivalent to a letter grade of B– (good or acceptable; Figure 2). Although usability was evaluated to be generally good, some areas for improvement were identified. Specifically, the scores were relatively low for utility (question 1), complexity (question 2), integration (question 5), unity (question 6), and satisfaction (question 9), indicating that the functional needs of the users were not sufficiently met and the method of use was not intuitive (Table 5).

**Table 5:** System Usability Scale evaluation results (N=5).

Item	Score (1-5), mean (SD; range)	Scaled score (0-100), mean (SD)
Total	3.02 (0.27; N/A^a^)	73.50 (11.40)
Utility (question 1)	3.80 (0.45; 3-4)	70.00 (11.18)
Complexity (question 2)	2.40 (0.55; 2-3)	65.00 (13.69)
Simplicity (question 3)	4.00 (0.00; 4-4)	75.00 (0.00)
Professionalism (technician support; question 4)	2.00 (0.71; 1-3)	75.00 (17.68)
Integration (question 5)	3.80 (0.84; 3-5)	70.00 (20.92)
Unity (question 6)	2.40 (0.89; 1-3)	65.00 (22.36)
Learnability (question 7)	4.40 (0.55; 4-5)	85.00 (13.69)
Convenience (question 8)	2.00 (1.00; 1-3)	75.00 (25.00)
Satisfaction (question 9)	3.80 (0.84; 3-5)	70.00 (20.92)
Professionalism (previous learning; question 10)	1.60 (0.55; 1-2)	85.00 (13.69)

^a^N/A: not applicable.

## Discussion

### Principal Findings

In this study, a formative evaluation was conducted of the usability of a tablet-based cognitive rehabilitation software to identify areas for improvement in the UI and UX. The combined use of CW and the SUS enabled the identification of both interaction-based usability problems and perceived UX among occupational therapists—the primary users of this software.

Key usability issues identified in this study, such as suboptimal keypad design, lack of instructional prompts, nonintuitive task termination mechanisms, and inconsistent iconography, reflect common design failures in medical software interfaces [21,22]. These issues were not merely aesthetic or functional flaws but directly influenced users’ ability to complete tasks efficiently and accurately. For example, the absence of clear visual cues for task completion or the ambiguous labeling of interactive elements (eg, the hint button) disrupted workflow continuity, increased cognitive burden, and raised the risk of data loss—factors that undermine both clinical reliability and therapeutic engagement. These findings align with those of previous studies emphasizing that poorly designed interfaces in medical device software can degrade user efficiency and negatively affect patient outcomes [23,24].

From a human factor perspective, many of the observed errors can be interpreted through the lens of cognitive load theory [25]. Inconsistent UI layouts and insufficient feedback mechanisms can impose extraneous cognitive load, diverting attention from therapeutic interaction toward interface management. In pediatric contexts, this can be particularly detrimental as delays or confusion in therapist-patient interaction may reduce children’s motivation and task adherence [24].

The software achieved an SUS score corresponding to a good or acceptable usability grade (B−); however, several dimensions—such as complexity, functional integration, and intuitiveness—scored below the average. These findings suggest a potential misalignment between the system’s interface design and the clinical workflows of end users. Consistent with previous research, a high overall SUS score does not necessarily indicate the absence of critical usability issues, as deficiencies in specific subdimensions may still reflect underlying problems related to workflow integration and task efficiency [26,27]. In contrast, higher ratings for learnability and professionalism indicate that, once users became familiar with the interface, they were able to operate the system with relative ease. This discrepancy underscores the importance of incorporating iterative usability testing and applying user-centered design principles throughout the software development life cycle to ensure both functional adequacy and clinical relevance [28,29].

On the basis of previous literature and these findings, several actionable design recommendations are proposed [27,30]: (1) redesign the size and placement of input interfaces to minimize use errors, (2) introduce guided workflows and clearly indicated start and termination controls to enhance procedural clarity and data integrity, (3) improve the visibility and semantic clarity of educational elements such as user manuals and functional icons, and (4) redesign interaction components—such as difficulty adjustment and hint functions—to follow a consistent and intuitive visual language that aligns with user expectations.

These recommendations highlight that formative evaluation should not focus solely on user satisfaction or interface aesthetics but should be systematically integrated with the software’s clinical business processes, including patient registration, cognitive assessment, therapy execution, and result review. From a clinical standpoint, improving task flow and feedback mechanisms within cognitive rehabilitation software can directly enhance therapeutic efficiency, reduce therapists’ cognitive workload, and increase patient engagement and adherence. Moreover, by aligning formative evaluation with the principles of the IEC 62366-1 standard and the International Organization for Standardization risk management standard 14971, this study demonstrates that usability engineering can serve as a practical framework for manufacturers seeking to design safer and more effective rehabilitation devices. Collectively, these findings emphasize the importance of integrating usability testing not as an isolated activity but as an iterative, evidence-based component of the medical software development process.

Despite its contributions, this study has several limitations. The evaluation involved a small and homogeneous sample of 5 occupational therapists from a single institution, which is appropriate for formative evaluation under the IEC 62366-1 standard but limits generalizability to broader clinical contexts or end users such as patients. Additionally, testing was conducted in a controlled simulation environment rather than during real-world therapy sessions, which may reduce ecological validity. Moreover, task scenarios were internally reviewed but not pilot-tested with end users, which could limit their realism and appropriateness. Finally, this study examined only therapists’ perspectives, excluding other user groups (eg, patients) who also interact with cognitive rehabilitation software.

Future studies should expand usability evaluations to include diverse user groups and real-world clinical settings, enabling longitudinal verification of software improvements and their impact on therapeutic outcomes. Furthermore, integrating objective usability measures—such as automated cognitive load assessments or eye tracking—could provide deeper insights into user interaction patterns and interface demands.

Overall, this study contributes empirical evidence supporting structured, user-centered design approaches for medical device software, particularly in cognitively demanding rehabilitation contexts. The findings provide practical guidance for developers and usability engineers seeking to optimize UI and UX design in alignment with clinical workflows and user requirements, thereby improving both safety and clinical effectiveness in digital cognitive rehabilitation interventions.

### Conclusions

This study conducted a formative evaluation of a tablet-based cognitive rehabilitation software applying CW and the SUS within the IEC 62366-1 framework. The results revealed critical usability challenges related to interface clarity, consistency, and information visibility—factors that directly affect user efficiency and the clinical reliability of software as a medical device.

In response to the identified issues, several targeted UI and UX improvement strategies were proposed, including resizing and repositioning the keypad, clarifying task initiation and termination processes, enhancing the visibility and labeling of instructional elements, and unifying the design of interaction features such as the hint and difficulty adjustment functions. These refinements are expected to reduce cognitive workload and support error-free operation.

Beyond individual design improvements, this study reinforces the importance of applying structured, standard-compliant usability engineering practices to medical and rehabilitation software. The integration of CW and the SUS proved effective in capturing both interaction-level issues and perceived usability gaps, underscoring the value of iterative, user-centered design processes during the early stages of development.

Future research should expand usability evaluations to real-world clinical environments and include direct feedback from end users. The adoption of objective usability measures such as eye tracking or use log analysis may further deepen insight into interaction behavior and inform next-generation interface design for digital therapeutics.

## Data Availability

The datasets generated or analyzed during this study are available from the corresponding author on reasonable request.

## Abbreviations

CW: cognitive walkthrough
IEC: International Electrotechnical Commission
NRC: National Rehabilitation Center
SUS: System Usability Scale
UI: user interface
UX: user experience
